# Hydrophilic polymer-stabilized porous composite membrane for water evaporation and solar desalination†

**DOI:** 10.1039/c9ra09667a

**Published:** 2020-01-14

**Authors:** Xiaoning Han, Linlin Zang, Shaochun Zhang, Tianwei Dou, Liang Li, Jian Yang, Liguo Sun, Yanhong Zhang, Cheng Wang

**Affiliations:** Key Laboratory of Functional Inorganic Material Chemistry (MOE), Heilongjiang University Harbin 150080 China wangc_93@163.com; School of Chemical Engineering and Materials, Heilongjiang University Harbin 150080 China sunliguo1975@163.com zhangyanhong1996@163.com; State Key Laboratory of Urban Water Resource and Environment, Harbin Institute of Technology Harbin 150080 China; Harbin Academy of Products Quality Inspection Supervision Harbin 150080 China

## Abstract

Solar steam generation is considered an effective and sustainable method for addressing freshwater shortages. However, several challenges to developing photothermal materials and improving evaporation performance currently exist. Herein, we designed a hydrophilic evaporator with double-layer structure by combining a hydrophilic polymer with three-dimensional porous carbon nanotube beads on a glass microfiber membrane. Poly(methacrylic acid) acted as a binder to stabilize the carbon-based photothermal layer along with continuously pumped water. The assembled carbon nanotube beads with porous structures not only harvested and converted light to heat but also provided available channels for fast vapor diffusion. An artificial tree evaporation configuration can effectively localize heat on the photothermal layer, which endowed the evaporator with a high evaporation rate of 1.62 kg m^−2^ h^−1^ with a solar-to-vapor energy conversion efficiency of 87% under 1 sun illumination. Meanwhile, excellent desalination performance and stable recycling test made the evaporator have great potential in practical applications.

## Abstract 2

A polymer-stabilized interfacial evaporator was designed with a porous structure, and exhibited excellent water evaporation and solar desalination performance.

## Graphical abstract

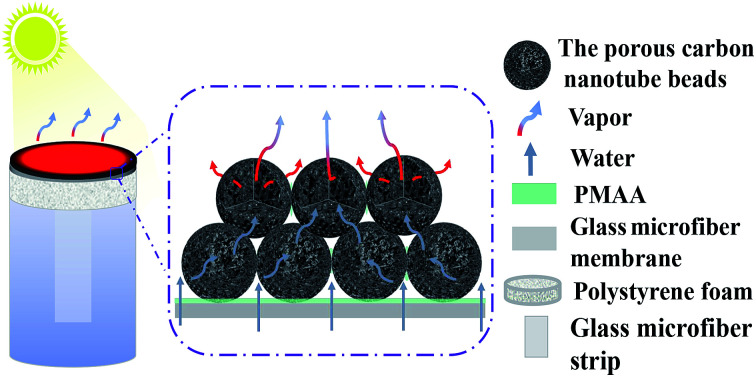

## Introduction

Water scarcity has become a severe problem around the world in the past decades because of population growth, industrial development and environmental pollution.^1–3^ Therefore, membrane separation processes based on reverse osmosis (RO) have been developed to produce clean water from seawater.^4^ However, sophisticated equipment, complicated pretreatment procedures, high energy consumption, and serious membrane fouling limit its application in remote inland areas and coastal cities.^5^ Recently, solar-driven interfacial steam generation (SISG) has attracted extensive attention as a promising and sustainable water treatment and desalination technology.^6–12^ Unlike traditional solar evaporation technologies, photothermal materials in the SISG system can efficiently harvest light and convert it to heat confined water at the interface instead of directly heating bulk water. This reduces heat loss and improves light-to-vapor conversion efficiency.^13–17^

At present, carbon-based photothermal materials are commonly used because of the advantages of their broadband light absorption, low cost, and large-scale preparation.^18–20^ Carbon nanotubes (CNTs), which have stable physical and chemical properties, good thermal stability and low specific heat capacity, have been particularly used as two-dimensional (2D) light absorbers in solar steam generation. For example, Hu's group prepared a wood-based evaporator by directly coating 2D CNTs on the surface, but this evaporator only achieved a high efficiency of 81% under 10 suns illumination.^21^ Weng *et al.* added beeswax into a mixture of CNTs and polydimethylsiloxane to construct an evaporator with a biomimetic superhydrophobic surface.^22^ Although this method can be widely applied to various substrates, the evaporation rate is less than 1.4 kg m^−2^ h^−1^ under 1 sun illumination and the solar-to-vapor conversion efficiency is only 82%. There may be two reasons for this ideal evaporation performance: (1) small pores formed by entangled and stacked 2D CNTs affected water vapor diffusion, or (2) insufficient contact between water and photothermal materials resulted in a low evaporation rate. It has been reported that optimizing the pore structure of carbon-based photothermal materials can effectively maximize light absorption and provide more channels for fast escape of water vapor.^23^ Therefore, a hierarchically porous structure and good water transportation are critical for improving the evaporation performance of CNTs-based evaporators.

Herein, we prepared a highly efficient solar evaporator consisting of a CNTs-based photothermal layer and a hydrophilic microfiber membrane. Three-dimensional (3D) porous carbon nanotube beads (PCNTBs) with uniform size can be tightly bound *via* a poly(methacrylic acid) (PMAA) coating and assembled on the surface of the support, thereby forming a stable PCNTBs/PMAA/glass microfiber (PCPG) composite membrane. The porous beads were fabricated by a microfluidic device using polystyrene microspheres as hard template. The abundant pores can confine the light in the beads, while providing channels for water transport and vapor diffusion. Meanwhile, hydrophilic PMAA can promote the efficient transport of water in the composite membrane. Besides, an artificial tree configuration was employed during the evaporation, which can efficiently improve the utilization and localization of the converted heat energy according to Mi and her co-authors' report.^5^ These features can enhance the evaporation performance of the PCPG composite membrane, making it a promising photothermal evaporator for solar desalination and water treatment.

## Experiment

### Materials

CNTs (diameter = 20–40 nm, length = 1–2 μm) were purchased from Shenzhen Nanotech Port Co., Ltd. Concentrated sulfuric acid, concentrated nitric acid, methyl acrylic acid (MAA), potassium persulfate and styrene monomer were purchased from Sinopharm Chemical Reagent Co., Ltd. Dimethyl silicone and *n*-hexane were purchased from Tianjin Fuyu Chemical Reagent Co., Ltd. The glass microfiber membrane was purchased from Whatman, UK and the average pore size was 1.2 μm. Poly(methyl acrylic acid) (PMAA, *M*_w_ = 310 000) was fabricated by free radical polymerization.^24,25^

### Preparation of the PCPG composite membrane

**Fig. 1:**
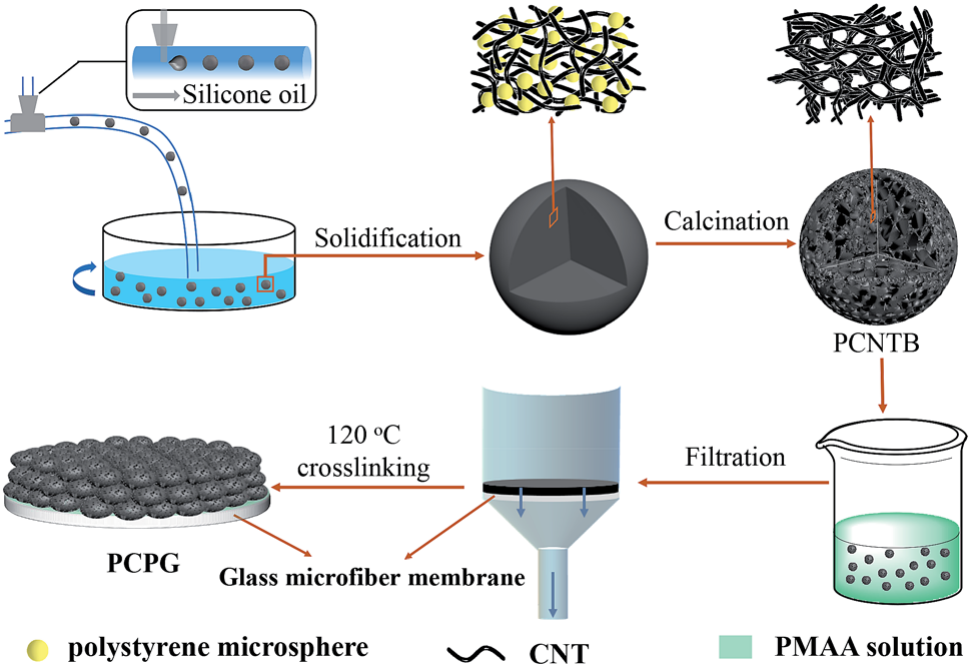
Schematic illustration of the preparation of the PCPG composite membrane.

Fig. 1 shows the preparation process for the PCPG composite membrane. First, the PCNTBs were fabricated *via* the hard template method using a modified microfluidic device.^26–28^ The detailed synthesis process of the PCNTBs was described in the ESI.† Next, 12 mg of the as-prepared PCNTBs was uniformly dispersed into 6 mL of a 4 wt% synthesized PMAA solution. The mixture was poured into a filter and the beads were deposited on a 2 cm-diameter glass microfiber membrane. After being dried at 70 °C for 20 min, the composite membrane was cross-linked in an oven at 120 °C for 1 h. The loading mass of PMAA and PCNTBs was 27 mg. For comparison, carbon nanotube beads (CNTBs) were prepared by the same steps as the PCNTBs, except using polystyrene microspheres. And the CNTBs/PMAA/glass microfiber (CPG) composite membrane was prepared by the same method as the PCPG.

### Solar steam generation experiments

In this work, we constructed an artificial tree evaporation configuration to perform the solar steam generation. Deionized (DI) water, saline water or seawater was put into a quartz container with a diameter of 2.1 cm and the container was wrapped by thermal insulation material. The circular sample (2 cm in diameter) was placed on a polystyrene foam with low thermal conductivity during evaporation. The simulated solar was used to illuminate the composite membrane vertically from the top, and the water mass loss was continuously recorded for 60 min. During the experiment, the surrounding temperature and humidity maintained at 23 °C and 13%, respectively. The surface temperature of the composite membrane was measured using an IR camera.

### Characterization

The morphology and structure were explored by using scanning electron microscope (SEM, S-4800). FTIR spectra were obtained with ADVANCE III, and all samples were prepared by potassium bromide pellet method. X-ray diffraction (XRD) patterns were tested by a Bruker D8 Advance with a Cu Kα radiation. The simulated sunlight source was a 300 W xenon lamp (Microsolar 300UV, Perfect light). The light intensity was calibrated by a photo radiometer (PL-MW2000, Perfect light). Meanwhile, the temperature was measured by an infrared radiation (IR) camera (FLIR One Pro). The absorption spectrum was recorded using an UV-vis-NIR spectrometer (Shimadazu, UV 2550). Contact angle measurement was performed using a contact angle meter (OCA20, Dataphysics).

## Results and discussion

### Characterizations of the PCNTBs

**Fig. 2:**
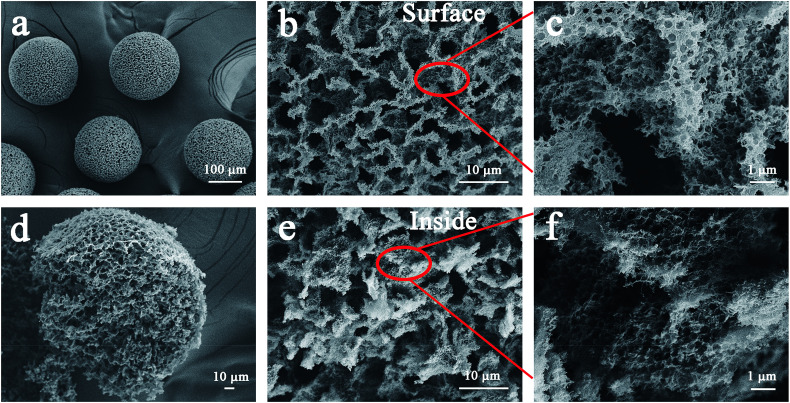
SEM images of (a) the PCNTBs, (b) and (c) the surface structure of the PCNTBs, (d) the broken PCNTBs, (e) and (f) the inside structure of the PCNTBs.

The CNTs after acid treatment still maintained the winding structure (Fig. S1†). The acidified CNTs with oxygen-containing groups indicated that they can uniformly disperse in composite droplets produced by the microfluidic device (Fig. S2†). Furthermore, in the solidification process, the acidified CNTs wrapped around the embedded hydrophilic polystyrene microspheres (360 ± 5 nm), resulting in a dense structure when water evaporating (Fig. S3†). It can be seen from Fig. S3c and d,† a large number of polystyrene microspheres randomly stacked. And the characteristic peaks of polystyrene microspheres and acidified CNTs are obviously observed in acidified CNTs/polystyrene beads (Fig. S4†). After high temperature calcination, polystyrene microspheres were removed from the composite beads as the hard template to form the porous structure throughout the beads (Fig. 2a–f). As shown in Fig. 2a, the acidified CNTs were made into 3D self-supporting beads with an average diameter of 200 ± 20 μm. Among them, assembled polystyrene microspheres resulted in the formation of submicron pores between the entangled CNTs (Fig. 2c and f), while larger pores were generated due to a large number of randomly stacked microspheres (Fig. 2b and e). The three-dimensionally structured PCNTBs possessed interconnected porous structure, therefore light can be multi-scattered in pores to promote light absorption.

Furthermore, in order to investigate the robustness of the PCNTBs, the beads were added to different shear flows to observe the spherical changes (Fig. S5†). It can be seen from the microscopy images that the PCNTBs both maintained good spherical morphologies after stirring in water for 15 min at different stirring speeds. The results demonstrate that the PCNTBs have good mechanical robustness, which is beneficial to the preparation of the composite membrane and the water evaporation experiment.

### Characteristics of the PCPG composite membrane

**Fig. 3:**
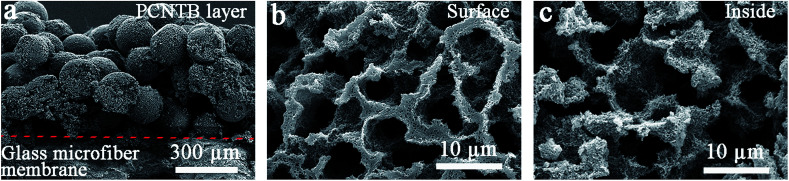
SEM images of (a) the cross-section view, (b) the surface structure and (c) the inside structure of the PCPG composite membrane.

The PCPG composite membrane was cut by a scissor for SEM characterization. The CNTs-based photothermal layer was deposited on the surface of the support to form the PCPG composite membrane following the filtration treatment (Fig. 3a). As shown in Fig. 3b and c, the interconnected porous structure of the surface and inside of the PCNTBs on the PCPG composite membrane was still maintained after filtration. The large pores in the porous beads provide conditions for the formation of water channels. Meanwhile, the interconnected porous structure is beneficial for providing channels for fast vapor diffusion.

**Fig. 4:**
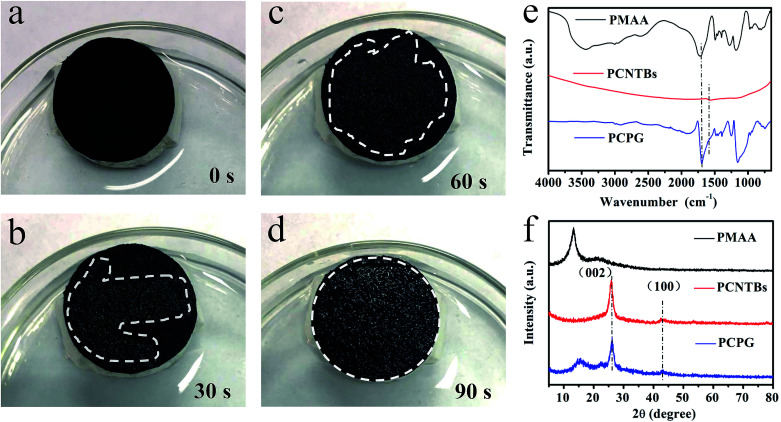
(a–d) Photographs of dynamic water absorption of the PCPG composite membrane; (e) FTIR spectra and (f) XRD patterns of PMAA, the PCNTBs and the PCPG.

The water transport capacity of the solar evaporator is critical for solar steam generation experiments. The water contact angle of the PCPG and the PCNTBs were tested, it was found that the PCPG composite membrane with PMAA exhibited excellent hydrophilicity (Fig. S6†). Fig. 4a–d show that the PCPG composite membrane was completely wetted in 90 seconds, demonstrating the excellent hydrophilicity and water transportation of the PCPG composite membrane. FTIR spectra indicate that the PCPG exhibits a strong peak at approximately 1730 cm^−1^, this is assigned to the carboxyl group of PMAA, which is absent in PCNTBs (Fig. 4e). XRD patterns show the PCPG retains the diffraction peaks of the CNTs and PMAA, centered at 26.1° and 15°, respectively (Fig. 4f). The above results proved PMAA was successfully mixed as the binder between the beads after the filtration.

**Fig. 5:**
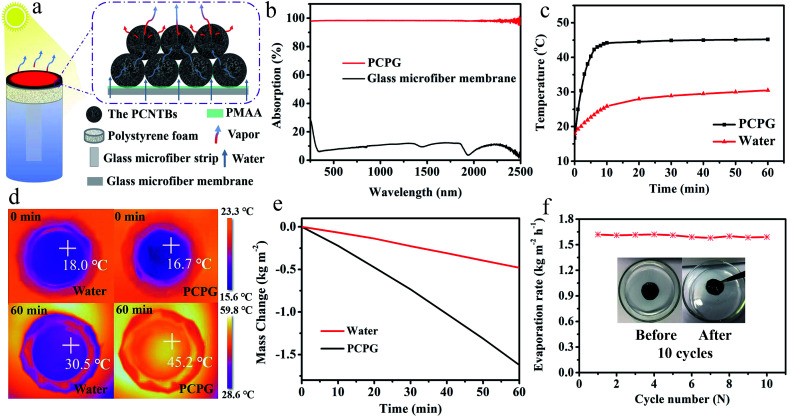
(a) Schematic illustration of the evaporator and evaporation process; (b) light absorption spectra of the PCPG composite membrane and the glass microfiber membrane in the wavelength range of 250–2500 nm; (c) surface temperatures of the PCPG composite membrane and water under 1 sun illumination for 1 h; (d) surface temperature changes of the PCPG composite membrane and water before and after 1 sun irradiation monitored by IR camera; (e) evaporation mass loss of the PCPG composite membrane and water under 1 sun illumination for 1 h; (f) solar evaporation rates of the same PCPG composite membrane under 1 sun illumination for 10 cycles with each sustained for 1 h. Insets are the pictures of the PCPG composite membrane before and after 10 cycles.

Fig. 5a shows a diagram of the solar evaporator, which was made of the PCPG composite membrane, polystyrene foam, a 2D glass microfiber strip and a glass container. In the artificial tree-like system, the glass microfiber acted as a trunk to transfer water from the bulk water to the composite membrane by capillary action. The composite membrane as leaves can localize heat to vaporize surrounding water bound by PMAA from the hydrophilic support, while the porous structure provides many channels for fast vapor diffusion. The light absorption spectra were used to determine the light absorption ability of the PCPG composite membrane. As shown in Fig. 5b, the light absorbance of the composite membrane can reach 98% in the wavelength range of 250–2500 nm, which much higher than that of the glass microfiber membrane. In contrast, the light absorption of the CPG (94%) showed a decreased intensity (Fig. S7†). These results indicate that the good light absorption of the PCPG could be attributed to the intrinsic absorption properties of CNTs and multiple scattering of light in the porous structure.^29^ The high light absorption made the surface temperature of the PCPG composite membrane rise to 44.2 °C in 10 minutes, while the surface temperature of water below 26 °C (Fig. 5c). After 1 h of illumination, the surface temperature of the composite membrane was 45.2 °C which was 14.7 °C higher than that of only water (Fig. 5d). Meanwhile, the temperature of the bulk water in the tree-like evaporation configuration was kept at 28 °C due to the presence of the thermal insulation. Therefore, it can be seen that the artificial tree configuration can effectively localize the converted heat on the photothermal layer, thereby realizing the interfacial water evaporation. In contrast, the direct evaporation of water remarkably led to energy waste. The PCPG composite membrane had a higher evaporation rate when the content of PCNTBs was 12.0 mg (Fig. S8†), which was 1.62 kg m^−2^ h^−1^ and about 3.4 times larger than that of pure water (Fig. 5e). In comparison, the evaporation rate of the CPG dropped to 1.43 kg m^−2^ h^−1^ (Fig. S9†). Moreover, solar-to-vapor conversion efficiency (*η*) was calculated according to the following equation:^5^
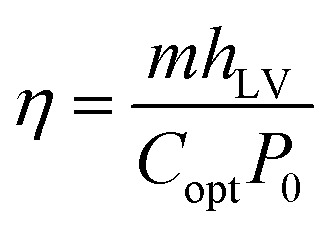
where *m* represents the mass flux subtracted by the evaporation rate under dark condition (0.32 kg m^−2^ h^−1^), *h*_LV_ is the total enthalpy of liquid–vapor phase change, *C*_opt_ is the optical concentration, and *P*_0_ is the solar illumination of 1 sun (1 kW m^−2^). The solar-to-vapor conversion efficiency of the composite membrane was calculated to be 87%. These results indicate that the evaporation performance of the composite membrane is higher than that of pure water, which may be attributed to its good light absorption capacity and water transport ability. Fig. 5f shows the performance of the PCPG composite membrane at 10 cycles under 1 sun illumination, in which each cycle sustained for 60 min. The evaporation rate of each cycle was maintained around 1.6 kg m^−2^ h^−1^. Meanwhile, as shown in insets, the morphology of the membrane had the excellent stability. These results indicate that the PCPG composite membrane can be recycled.

**Fig. 6:**
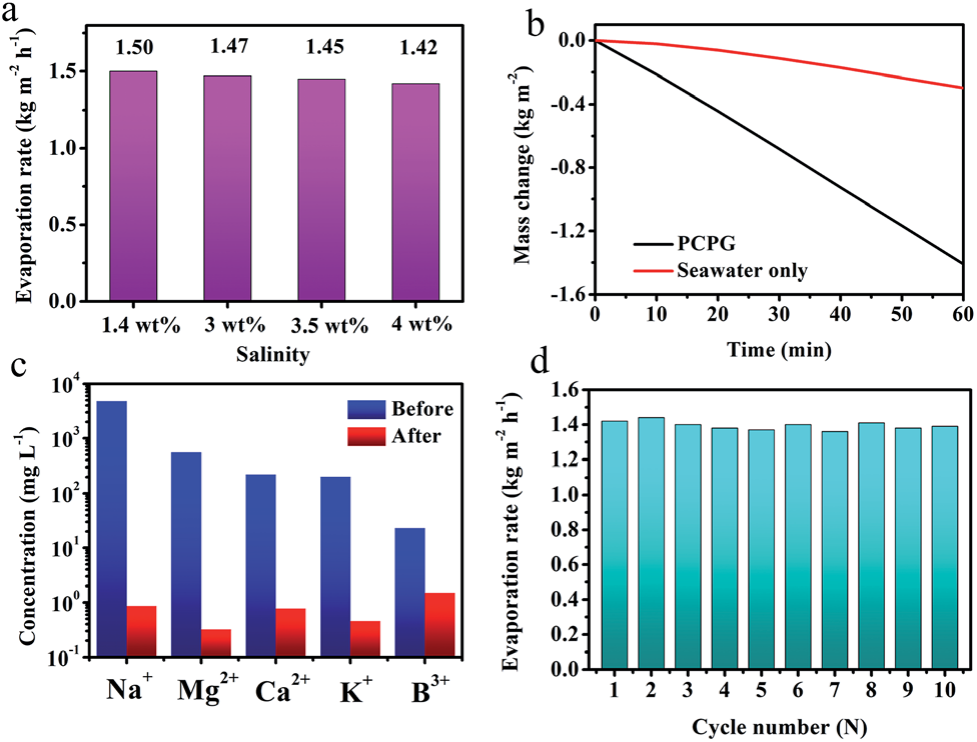
(a) The evaporative rate of the PCPG composite membrane under different concentrations of NaCl solutions under 1 sun illumination for 1 h. (b) Evaporation mass loss of seawater with the PCPG composite membrane and without any absorbent under 1 sun illumination for 1 h. (c) The measured concentrations of the five major ions before and after desalination of actual seawater samples. (d) Solar evaporation rates of seawater with the PCPG composite membrane under 1 sun illumination for 10 cycles with each sustained for 1 h.

Besides, we investigated the solar desalination performance in the simulated saline water and actual seawater using the tree-like evaporator. As we known, the vapor pressure of saline water is lower than the pure water due to the presence of various salt ions. At the same time, the viscosity of the solution becomes large as the salt concentration increases concentration increases. Therefore, the evaporation rate in the saline water is a lower than that in the pure water without ion interference. During the vaporization of saline water with different NaCl concentrations, the evaporation rate did not significantly change, which was maintained at around 1.45 kg m^−2^ h^−1^ (Fig. 6a). These results indicate that the PCPG composite membrane still maintains the freshwater production capacity even in saline water. For actual seawater (seawater sample average salinity ≈ 3.5 wt%, from Bohai Sea, China), the evaporation rate using the tree-like configuration was as high as 1.41 kg m^−2^ h^−1^, which was 4.6 times faster than that direct evaporation of seawater (Fig. 6b). And concentrations of Na^+^, Mg^2+^, K^+^, B^3+^ and Ca^2+^ in the product water were much lower than the initial concentration in seawater, which meet WHO's water quality requirements for drinking water quality (Fig. 6c). These results demonstrate that the PCPG composite membrane has prominent desalination performance. Moreover, recycling stability of the PCPG composite membrane was proved by a multiple cycle evaporation test in the actual seawater. As shown in Fig. 6d, the evaporator had a stable evaporation rate of approximately 1.4 kg m^−2^ h^−1^ during 10 cycles of recycling.

## Conclusions

We successfully prepared a hydrophilic evaporator with a PMAA/PCNTBs photothermal layer. Under 1 sun illumination, it exhibits a high evaporation rate of 1.62 kg m^−2^ h^−1^ and a solar-to-vapor conversion efficiency of 87%. The unique 3D porous structure of the PCPG composite membrane enables it to have a high light absorption and vapor escape channels. Meanwhile, the porous structure and hydrophilic PMAA can promote the transport of water, thereby improving the water evaporation efficiency. Furthermore, the excellent desalination performance and stable reusability make it a promising evaporator for solar desalination and water treatment.

## Conflicts of interest

There are no conflicts to declare.

## Supplementary Material

RA-010-C9RA09667A-s001
