# Supplementary material for: Hydrophilic polymer-stabilized porous composite membrane for water evaporation and solar desalination
Source: RSC Adv. 2020 Jan 14;10(5):2507–12. doi: 10.1039/c9ra09667a (PMC9048818; doi:10.1039/c9ra09667a)
Supplement: RA-010-C9RA09667A-s001 [file RA-010-C9RA09667A-s001.pdf]

Electronic Supplementary Material (ESI) for RSC Advances.
This journal is © The Royal Society of Chemistry 2020

Supporting information

Hydrophilic Polymer-Stabilized Porous Composite Membrane for Water Evaporation and Solar Desalination

Xiaoning Han[a,b], Linlin Zang[c], Shaochun Zhang[b], Tianwei Dou[a], Liang Li[b], Jian Yang[d], Liguo Sun[b*],Yanhong Zhang[b], Cheng Wang[a*]

[a] Key Laboratory of Functional Inorganic Material Chemistry (MOE), Heilongjiang University, Harbin, 150080, China. E-mail: wangc_93@163.com

[b] School of Chemical Engineering and Materials, Heilongjiang University, Harbin, 150080, China. E-mail: sunliguo1975@163.com, zhangyanhong1996@163.com

[c] State Key Laboratory of Urban Water Resource and Environment, Harbin Institute of Technology, Harbin, 150080, China

[d] Harbin Academy of Products Quality Inspection Supervision, Harbin, 150080, China

1. Preparation

1.1 Acid-oxidation treatment for CNTs

A volume ratio of 3:1 to a mixture of concentrated sulfuric acid and nitric acid was added to the flask, followed by the addition of 5 g of CNTs. The mixture was then heated to reflux at 60 ^{o}C for 3 h with stirring. After completion of the reaction, the resulting suspension was cooled to room temperature, and then washed repeatedly and dialyzed for several days. Finally, water-dispersed CNTs powders were obtained after drying at 80 ^{o}C. CNTs (diameter=20~40 nm, length=1~2 μm) were purchased from Shenzhen Nanotech Port Co., Ltd. Concentrated sulfuric acid and concentrated nitric acid were purchased from Sinopharm Chemical Reagent Co., Ltd.

1.2 The synthesis of polystyrene microspheres.

Polystyrene microspheres were synthesized by soap-free emulsion polymerization.[1] A typical synthesis of 360 nm polystyrene microspheres: 500 mL of water, 150 mL of styrene monomer and 5 mL of methacrylic acid were added to a three-necked flask, heated at 80 °C and stirred at 300 rpm. After the reaction system was refluxed for 3 min, 0.5 g of a potassium persulfate initiator was added. After reacting for 3 h, the product was repeatedly centrifuged to obtain a product.

1.3 Preparation of porous carbon nanotube beads (PCNTBs)

The continuous phase was dimethyl silicone oil, and the dispersed phase was prepared as follows: 4.52 g acidified CNTs solution (4.42 wt.%) and 6 g polystyrene

microspheres solution (10 wt.%) were mixed under strong magnetic stirring and ultrasonic treatment. The pushing speed ratio between the dispersed and the continuous phase was adjusted to 0.8: 150. Then acidified CNTs/polystyrene droplets were received by a slowly moving vessel which contained a certain amount of dimethyl silicone oil in advance and solidified at 60 °C for 12 h. Subsequently, the beads were repeatedly washed with n-hexane and dried. At last, the beads were heated at 700 °C for 2 h under nitrogen atmosphere to obtain the PCNTBs.

2. Characterization

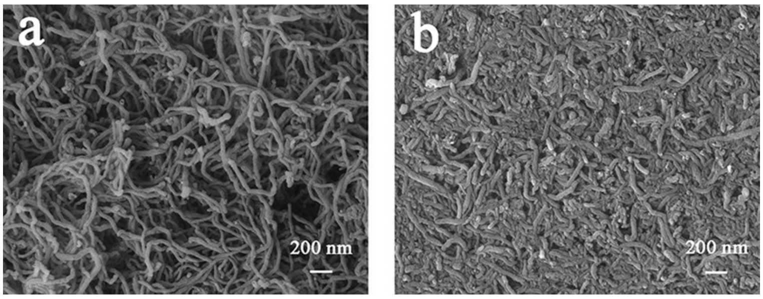

Fig. S1 SEM images of pristine CNTs (a) and acidified CNTs (b).

In order to form the evenly structured droplets, CNTs had been acidified to make it super hydrophilic. The peak at 1574 cm^{-1} occurring in pristine CNTs and acidified CNTs was attributed to stretching vibrations in the sp^2 region of CNTs. The acidified CNTs showed a peak at 1705 cm^{-1} belonging to the C=O, indicating the presence of oxygen-containing groups (Fig.S2a). As shown in the XRD patterns in Fig.S2b, the diffraction peak appeared at 26.1° and 43.5° related to the (002) and (100) planes of a graphite-like structure, respectively. These results indicated that acidified CNTs can uniformly disperse in composite droplets.

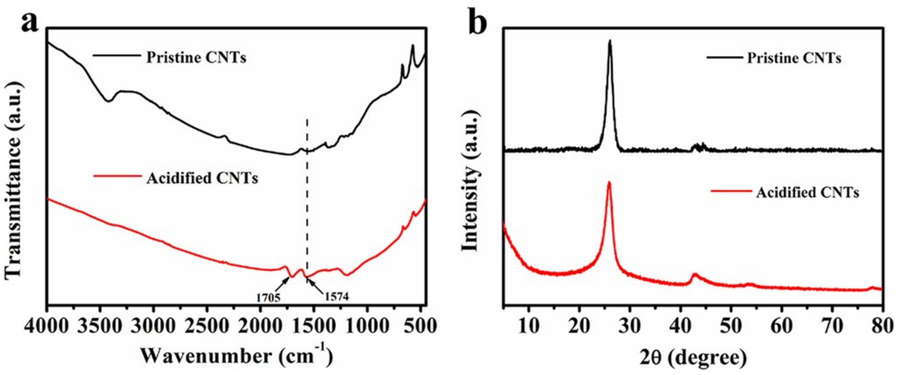

Fig. S2 FTIR spectra (a) and XRD patterns (b) of pristine CNTs and acidified CNTs.

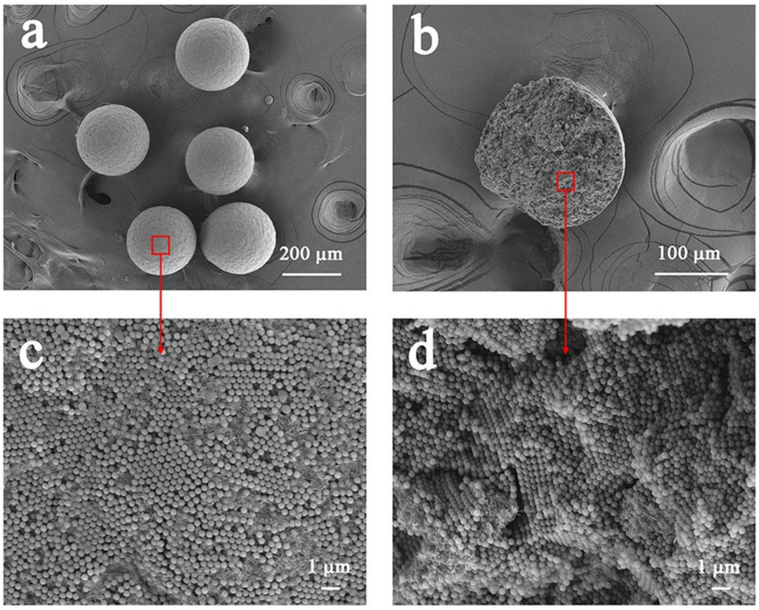

Fig. S3 SEM images of acidified CNTs/polystyrene beads after solidification: (a) acidified CNTs/polystyrene beads, (b)the section of a broken bead, and magnified images of (c) the surface structure and (d) the inside structure.

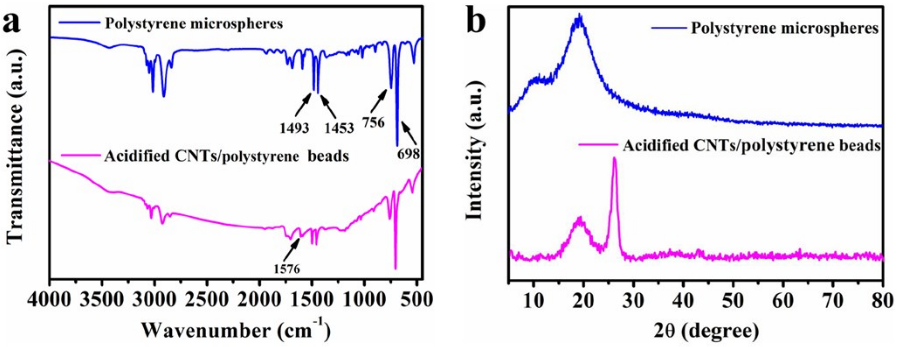

Fig. S4 FTIR spectra (a) and XRD patterns (b) of polystyrene microspheres and acidified CNTs/polystyrene beads.

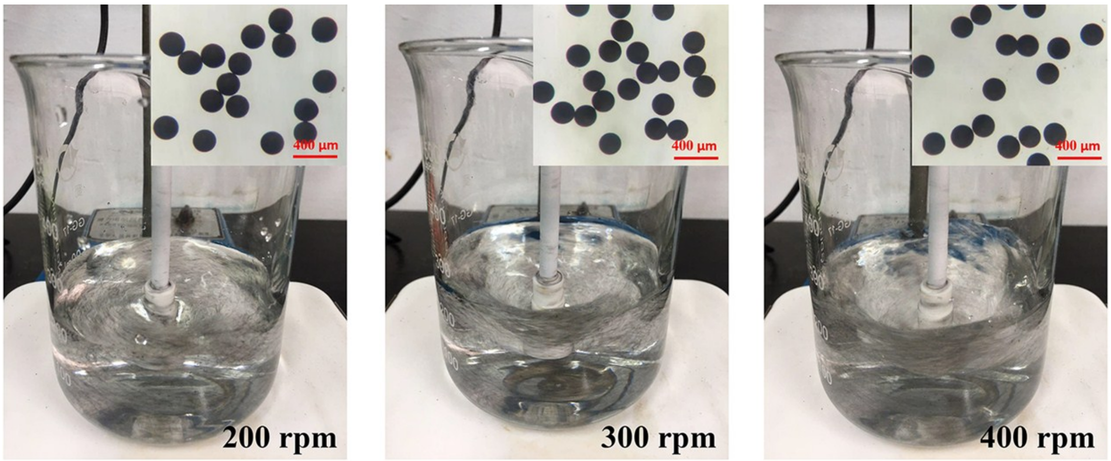

Fig. S5 Digital photos of the PCNTBs in pure water system under continuous stirring for 15 min at different speed (200, 300 and 400 rpm) and the corresponding microscope photographs.

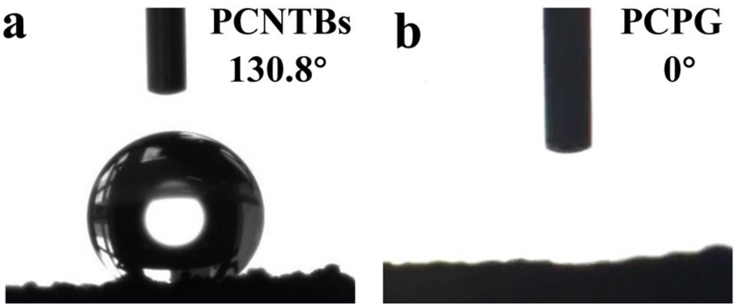

Fig. S6 Contact angle measurements of water on the PCNTBs (a) and the surface of the PCPG composite membrane (b).

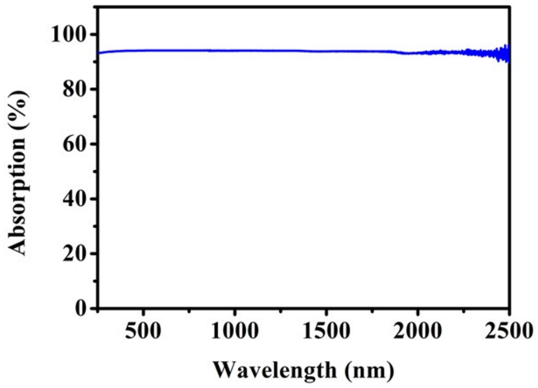

Fig. S7 Light absorption spectra of the CPG composite membrane in the wavelength range of 250-2500 nm.

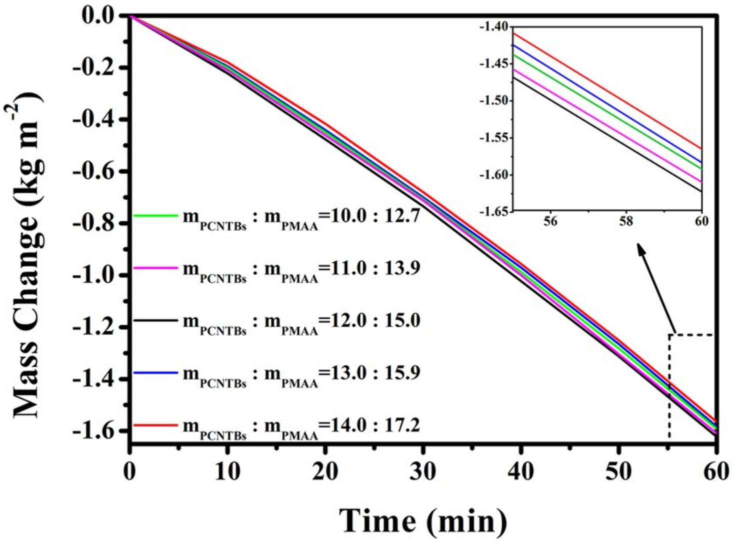

Fig. S8 Evaporation rates of the PCPG composite membranes with different mass ratios of the PCNTBs and PMAA under 1 sun illumination for 1 h.

The PCPG composite membranes with different mass ratios of the PCNTB and

PMAA were prepared by the same method, and their evaporation rates were compared. Fig. S8† indicated the mass ratio of 12.0 : 15.0 is optimal in this work.

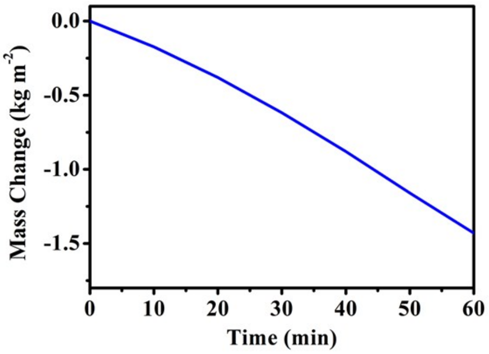

Fig. S9 Evaporation mass loss of the CPG composite membrane under 1 sun illumination for 1 h.

Reference:

1 Z.Z. Gu, H. Chen, S. Zhang, L. Sun, Z. Xie, Y. Ge, Eng. Asp., 2007, 302, 312-319.
